# Allosteric regulation of proteolytic machines unveiled by the synergy between cryo-EM and solution NMR spectroscopy

**DOI:** 10.1042/BCJ20253239

**Published:** 2025-08-26

**Authors:** Madison Turner, Robert W. Harkness, Zev A. Ripstein, Rui Huang, Siavash Vahidi

**Affiliations:** 1 Department of Molecular and Cellular Biology, University of Guelph, Ontario N1G 2W1, Canada; 2 Department of Chemistry, University of Manitoba, Manitoba R3T 2N2, Canada; 3 Department of Chemistry, University of Guelph, Ontario N1G 2W1, Canada

**Keywords:** AAA proteins, allosteric regulation, cryo-electron microscopy, NMR spectroscopy, protein dynamics, serine proteases

## Abstract

Mechanistic studies of biomolecular machines involved in intracellular protein degradation—such as the caseinolytic protease P, ATPases associated with diverse cellular activities (AAA+) motors, and the high-temperature requirement A family of enzymes—are of great interest as they are implicated in a host of human diseases. The function of these systems is dependent on both their fine-tuned three-dimensional structure and the conformational dynamics that modulate this structure. Their large sizes, inherent conformational plasticity, and oligomeric heterogeneity dictate that their mechanism of action cannot be deciphered by any one method. Synergistic application of methyl-transverse relaxation optimized spectroscopy (methyl-TROSY), nuclear magnetic resonance (NMR), and single-particle electron cryomicroscopy (cryo-EM) has uniquely positioned researchers to tackle the outstanding questions in this area of structural biology. Cryo-EM enables structural characterization and modeling of the large and conformationally heterogeneous complexes involved in protein degradation, while methyl-TROSY NMR enables monitoring structural transitions and conformational dynamics of these systems in response to various stimuli in solution at atomic resolution. This review highlights how combining these two approaches offers a distinct and powerful means to unravel allosteric pathways within complex, multipartite biomolecular machines.

## Proteostasis and the proteolytic machinery

Protein turnover is a fundamental homeostatic mechanism indispensable for all organisms [1]. Intracellular proteolysis co-ordinates numerous vital cellular processes, such as cell cycle progression, differentiation, transcriptional regulation, immune surveillance, antigen processing, signaling cascades, endocytosis, metabolic regulation, and the maintenance of protein integrity [2–5]. Given the broad spectrum of functions, proteolytic pathways exhibit precise specificity to ensure that cellular proteins are held at the correct concentrations, kept in optimal conformations, and are correctly localized [6,7]. Disruptions within these finely tuned degradation systems are increasingly found to underlie various human pathologies, notably cancers [8–14] and neurodegenerative disorders [15–19]. Furthermore, targeting the protein degradation machinery of pathogenic organisms has emerged as a promising therapeutic strategy against antimicrobial-resistant infections [20–30]. The essential roles played by proteolytic systems in cellular health and disease underscore significant therapeutic potential; however, exploiting these pathways therapeutically requires detailed mechanistic insights into their structures, dynamics, regulatory mechanisms, and substrate interactions.

Intracellular proteolysis must be tightly regulated, as promiscuous engagement of substrate would result in spurious protein degradation and subsequently lead to cell death. To mitigate this risk, evolution has brought forth proteases with compartmentalized active sites where proteolysis occurs [31–38]. A prominent example is the AAA+ family of proteases, including the Clp proteases, HslUV, Lon, FtsH, and the 26S proteasome, which rely on co-ordinated assemblies of regulatory and catalytic components. In these systems, substrate recognition and delivery are managed by a regulatory particle (RP), either directly or via adaptor proteins that bind defined degradation signals (degrons) such as ubiquitin or SsrA tags [5,31,39,40]. Upon recruitment, substrates are mechanically unfolded and translocated through the RP’s central channel in a ‘hand-over-hand’ fashion powered by ATP binding and hydrolysis cycles conserved across AAA+ motors [41]. The unfolded polypeptide is then fed into a sequestered proteolytic chamber for degradation.

Beyond the AAA+ family of proteases, members of the high-temperature requirement A (HtrA) family have also been shown to have chamber-like features [42]. These trimeric enzymes can self-assemble under cellular stress conditions into homomeric cages consisting of trimer building blocks that encapsulate target substrates [42,43]. This promotes the processive proteolysis of substrates, which occurs in an ATP-independent manner via a ‘hold-and-bite’ mechanism [44]. Many HtrA family members also act as chaperones, allowing them to switch between protective and degradative roles depending on cellular needs [45–47]. This functional duality is underpinned by substantial structural plasticity that enables HtrA proteins to dynamically sense and respond to environmental cues. Therefore, while AAA+ protease complexes rely on the co-ordinated activities of multiple proteins and molecular motors, HtrA proteins function via an adaptive structural response within identical subunits to carry out different cellular activities. In both cases, effective proteolysis depends on finely tuned co-operative and allosteric dynamics across multimeric assemblies [37,44,48–52].

Allosteric enzymes operate on rugged energy landscapes that give rise to a dynamic ensemble of conformations, each associated with distinct levels of activity [53–56]. These conformers coexist in equilibrium and interconvert at rates governed by the energy barrier separating them [53–55,57]. Binding of allosteric effectors, either substrates or ligands that associate with distal non-catalytic sites, reshapes the energy landscape and shifts the distribution of conformational states, thereby modulating enzymatic function [53–55]. These allosteric effects often propagate across subunits and long distances, enabling conformational dynamics to finely tune responses to environmental stimuli and regulate biological function [53,57–59]. Indeed, AAA+ proteases undergo conformational change in response to various stimuli, including the binding and hydrolysis of nucleotides and substrate engagement [60], as well as interaction with various activators and adaptors [61–64]. Similarly, the binding of substrates influences the assembly and proteolytic function of HtrA family enzymes as noted above [44,52,65,66]. Structural plasticity is thus an integral component to the function of these protease systems and must be carefully considered when investigating the mechanisms that underpin their activity.

The structural heterogeneity and large size of AAA+ and HtrA proteases make probing their conformational dynamics a challenge for many structural and biophysical tools. Methods such as X-ray crystallography have provided important atomic-resolution insights, but capturing the full range of conformational states sampled during the activity cycle has remained difficult. Crystal structures typically represent a single, often ground-state conformation, while other solution-based techniques that accommodate heterogeneity often report ensemble-averaged data that can obscure key mechanistic features. With the widespread adoption of electron cryomicroscopy (cryo-EM), the number of available structures, and consequently our understanding of these systems, has expanded dramatically. Major advances have originated from integrative approaches that resolve both static architectures and dynamic transitions, revealing the co-ordinated molecular motions underlying protease function. In this review, we focus on the synergistic application of cryo-EM and nuclear magnetic resonance (NMR) spectroscopy in the study of oligomeric proteases, which has led to new insights that neither approach could provide alone. We illustrate this principle through several case studies in which structural heterogeneity and conformational dynamics within compartmentalized protease systems were uniquely resolved through this combined approach.

## Capturing structural heterogeneity and dynamics: combining cryo-EM and NMR

**Figure 1:**
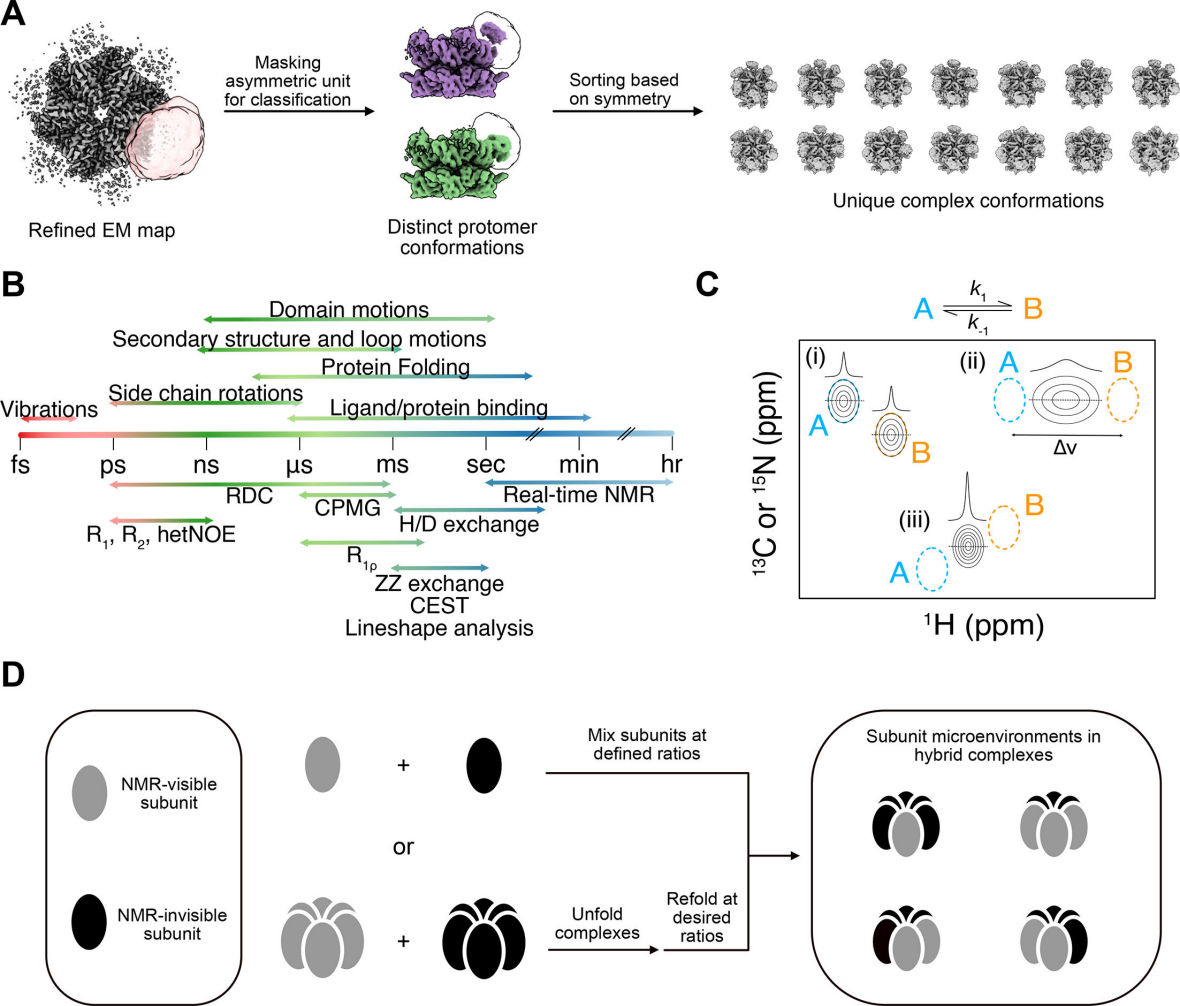
Capturing structural heterogeneity and conformational dynamics (**A**) Sample cryo-EM classification scheme for homo-oligomeric complexes. Refined cryo-EM density maps often contain averaged information on multiple conformations (left). Masking regions of interest combined with classification schemes can help sort out distinct conformations (middle and right) and provide information on allosteric changes across a complex by correlating neighboring motions such as domain movements; (**B**) timescales of the biologically relevant dynamic processes in proteins (above the timescale bar) and those that can be probed by various NMR experiments (below the timescale bar); (**C**) schematic of a 2D heteronuclear NMR spectrum of a biomolecule that exchanges between a pair of conformational states, A and B. Depending on the rate of exchange *k*_ex_ (*k*_ex_ = *k*_1_ + *k*_−1_) in comparison with the chemical shift difference between the two conformers (Δν), the spectrum may contain multiple sets of peaks, each corresponding to a conformer (i, *k*_ex_ << Δν), or a single set of peaks with broad (ii, *k*_ex_ ≈ Δν) or narrow (iii, *k*_ex_ >> Δν) linewidths; and (**D**) schematic representing the possible microenvironments of an NMR-active subunit in a hybrid complex when only considering the neighboring subunits, as prepared through mixing experiments. Note that when considering subunit microenvironments, only a subset of the total number of possible combinations of oligomers need be considered. Panel (**A**) adapted from ref [74] with permission.

Cryo-EM has proven especially powerful in shedding light on the structural motions of proteases through its ability to provide snapshots of multiple coexisting conformational states [36,37,67]. While pervasive structural heterogeneity still poses a challenge, computational advances in cryo-EM image processing have, however, in many cases enabled the identification and classification of coexisting states and facilitated the reconstruction of conformations beyond the ground state [68–70]. As cryo-EM centers on obtaining many images of single particles for analyses, it is well suited to heterogeneous samples and enables the analysis of diverse structural species that are likely to reflect the ensemble observed in solution [71–73] (Figure 1A). In addition to the classification and structural mapping of distinct sub-states, innovations in image analyses have led to the ability to characterize continuous protein ‘breathing’ motions [75,76]. By capturing structural diversity, including low-resolution features in flexible regions, cryo-EM can provide a holistic view of the conformational states populated within an energy landscape. With the rapidly increasing number of high-resolution structures solved by cryo-EM, there is great interest in using these to understand how molecular dynamics translate into function. While cryo-EM can sometimes provide information on the fractional populations of different states, even with large particle counts, it can be difficult to reveal lowly populated structures. Furthermore, it does not report on the rates at which conformations interconvert. Limitations in local map resolution can also create obstacles by preventing the tracing of allosteric pathways such as those within the protease assemblies considered herein. There is, therefore, a need for a separate approach to address this gap.

Solution NMR spectroscopy is exceptionally suited to meet this challenge through its ability to simultaneously inform on the parameters governing an energy landscape and the structures of the biomolecules within them with atomistic and quantitative detail. NMR is sensitive to biomolecular dynamics over a wide range of timescales (ps to >s) that are associated with distinct types of structural fluctuations (Figure 1B). While many of these experiments are traditionally applied to smaller protein systems, experiments such as ^1^H/^13^C chemical exchange saturation transfer [77,78], multiple-quantum Carr-Purcell-Meiboom-Gill (CPMG) relaxation dispersion [79], and forbidden [80] coherence experiments have been specifically developed to extend NMR-based dynamic studies to larger protein complexes [81]. Those occurring on the microsecond to millisecond timescale often underpin key functional processes such as folding, substrate binding, and catalysis [82–84]. With the abundance of spin-relaxation experiments that have been developed, it is now relatively routine to characterize these motions and identify ‘hidden’ conformations that may be populated as little as 1%, yet these are the active form of an enzyme [85–88]. In addition, monitoring chemical shift perturbations (CSPs) as a function of a perturbant in a titration series (e.g. pH, ligand concentration, mutation, etc.) provides rich kinetic and thermodynamic information (Figure 1C). Biomolecular structural heterogeneity may manifest in these experiments as peak multiplicity (e.g. >1 ligand-bound peak indicating multiple bound conformations) or peak broadening depending on the timescales of the structural transitions relative to the chemical shift differences between the conformations at hand. Furthermore, allosteric pathways can be traced through the introduction of mutations at substrate recognition interfaces distal to the active site by monitoring CSPs. Of particular importance, the development of methyl-transverse relaxation optimized spectroscopy (methyl-TROSY) has greatly expanded the upper limit of molecular sizes that can be investigated by solution NMR spectroscopy, enabling the study of megadalton-sized complexes [89,90]. Since methyl groups are widely found in hydrophobic cores, interaction interfaces, and active sites of enzymes, they serve as sensitive site-specific probes of local structure and dynamics. There are also a multitude of advanced isotope labeling approaches to introduce, for example, single ^19^F and ^13^CH_3_ probes in regions of interest, circumventing the issue of spectral overlap that is sometimes encountered when interrogating large protein assemblies [91,92]. Collectively, the diverse toolkit offered by NMR allows detailed characterization of protein dynamics and the mapping of functional states of enormous complexes in solution with exquisite detail. A particularly powerful system where this has been demonstrated is the proteasome. Methyl-based NMR has been successfully applied to characterize conformational dynamics within the proteasome across a range of timescales [92–101]. Nevertheless, these experiments present challenges, particularly in the context of large protein complexes, where spectral overlap and line broadening can complicate data interpretation. Resonance assignment via mutagenesis is often labor-intensive and costly. In such cases, high-resolution structural data, in combination with NOE-based distance restraints, can facilitate more efficient and confident assignment of resonances [102]. The complementarity of cryo-EM and NMR spectroscopy [103], therefore, provides a robust means of probing conformational heterogeneity and dynamics within large biomolecular complexes.

This combined approach becomes even more powerful when applied to the study of co-operativity within protein complexes, which can be accomplished by exploiting subunit mixing, or ‘doping’ experiments which will be detailed below (Figure 1D). ‘Subunit mixing’ studies refer to experiments in which two subunit types (e.g. variant and wildtype (WT) subunits, denoted Type A and Type B in what follows, Figure 1D left) are combined to form hybrid complexes (Figure 1D middle and right). The two subunits are first expressed and purified separately before being mixed at defined ratios and allowed to oligomerize. Depending on the assembly mechanism, unfolding and refolding steps may be required. This results in a mixture of complexes with varying compositions, the populations of which can be controlled experimentally and calculated using mathematical modeling based on the binomial theorem, assuming random subunit mixing, as we previously showed [104,105]. While this approach was originally used to study allosteric regulation of activity in AAA+, HtrA, and related systems [66,104,106–108], pairing it with cryo-EM and NMR can reveal additional structural insights related to allostery. For NMR studies, selective isotopic labeling allows monitoring of signals from one subunit type within an assembly in which the other form of subunit is not isotopically labeled, in other words, NMR ‘invisible’. If the allosteric effect from only the nearest neighbors is considered, there are a total of four microenvironments for a given subunit as part of a closed oligomer: (1) sandwiched by a pair of Type A or Type B subunits on both sides, (2) having the clockwise neighbor being Type A subunit and the other being Type B, or having the clockwise neighbor being Type B subunit and the other being Type A (Figure 1D). Each microenvironment can then be explored by adjusting the mixing ratio, which modulates the relative populations of each state, and the resulting NMR spectra reveal how local environments influence conformational equilibria. Cryo-EM complements this by directly visualizing conformational outcomes of co-operative interactions across subunits [74]. Taken together, these approaches provide data for correlating structure and dynamics that inform on allosteric mechanisms within protein assemblies. In the following section, we review three large proteolytic systems where the coupling of NMR and cryo-EM has provided unique insights on protein dynamics and regulation. In these examples, the utility of these techniques is exemplified by the complementary of the data and the ability of these methods to inform one another in the process.

## A combined approach—Studies of dynamic proteostasis machines

### p97

p97, also known as VCP or Cdc48 in yeast, is a conserved eukaryotic AAA+ enzyme that participates in a variety of cellular functions related to proteostasis [109]. In the ER-, mitochondrial-, and nuclear chromatin-associated degradation pathways, p97 works to remove damaged or misfolded proteins from membrane structures or large complexes, thereby allowing for their degradation by the 26S proteasome in the cytoplasm [110–113]. Outside of participating in protein quality control, p97 also functions as a chaperone or segregase, with its activity and cellular function regulated by a diverse array of adaptor proteins [114]. These adaptor proteins also modulate substrate specificity, enabling p97 to recognize a wide variety of cellular targets. Consequently, p97 plays a central role in cell signaling via targeting regulatory proteins for degradation or conversely releasing them from larger structures where they are sequestered. As such, mutations that affect p97 function can have severe physiological consequences. In humans, mutations of p97 have been implicated in neurodegenerative diseases, including an autosomal dominant multisystem proteinopathy disorder (MSP1, also called IBMPFD) [115,116], vacuolar tauopathy [117], and amyotrophic lateral sclerosis [118]. Due to its importance in cellular homeostasis, p97 has been identified as a possible drug target for various diseases [119]. Yet, the development of p97-specific therapeutics requires a detailed understanding of structure/function relationships in both the WT and mutant p97 proteins. Notably, the conformational dynamics associated with ATP hydrolysis and adaptor binding have consistently been identified as key regulators of p97 function [110,111,120]. In principle, compounds that target the structural components involved in these fluctuations could be used to restore p97 function in diseases where they are disturbed.

**Figure 2:**
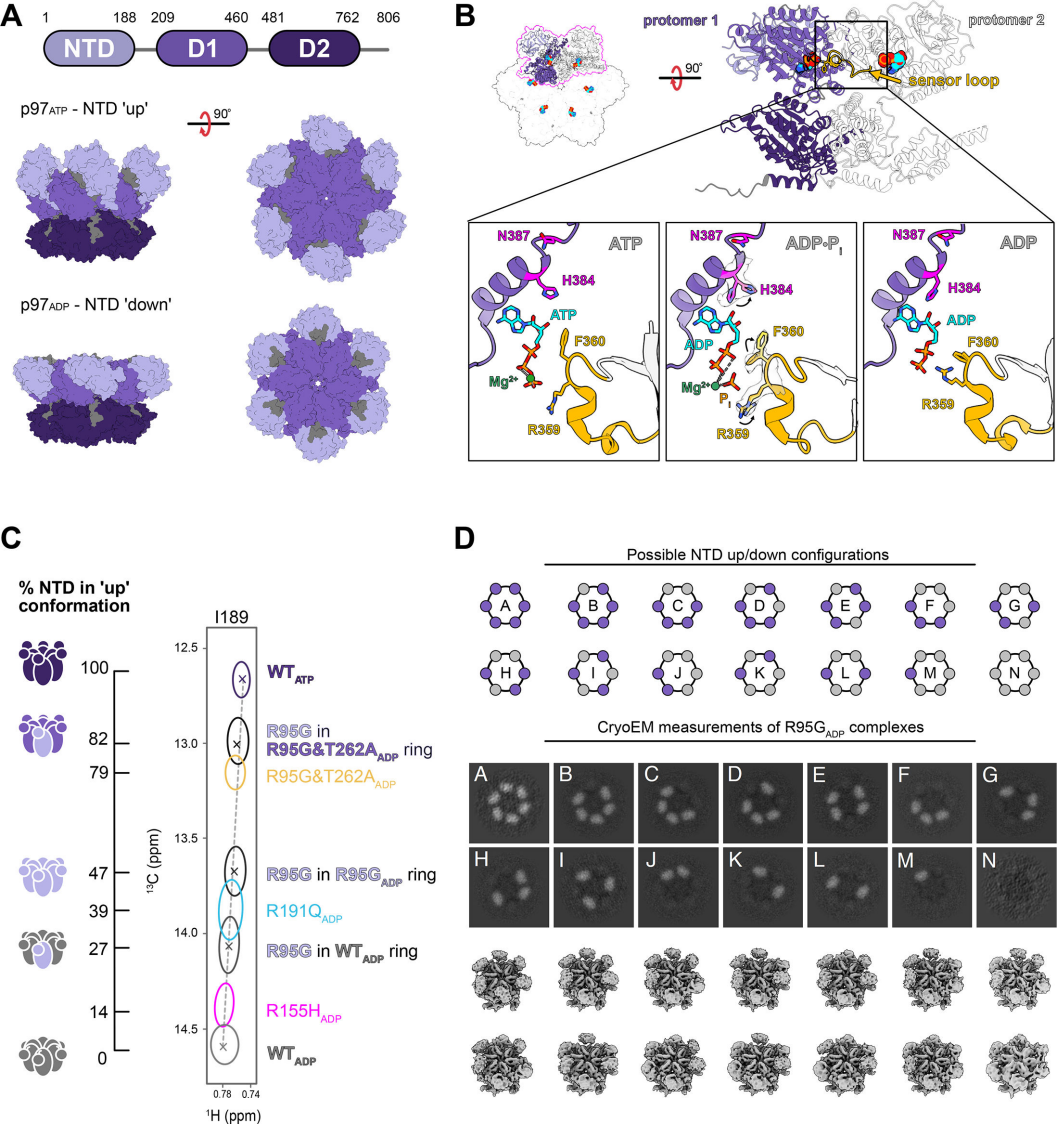
Synergistic methyl-TROSY NMR and cryo-EM studies of p97 NTD dynamics. (**A**) (Top) Domain arrangement of a p97 monomer. The NTDs of p97 adopt an ‘up’ conformation in the ATP state (middle) and a ‘down’ conformation in the ADP state (bottom) (PDB ID 5FTN [121]); (**B**) cryo-EM-identified rotamer shifts within the p97 active site upon ATP hydrolysis that mediate an electrostatic network responsible for the NTD conformational state [PDB ID 7LMY for ATP-bound [122], 8OOI for ADP-P_i_-bound [120], 5FTK for ADP-bound [121]]; (**C**) chemical shifts of NMR methyl probes, such as I189 shown schematically here, provide the fractional population of NTDs in the ‘up’ or ‘down’ conformation (**D**) 14 unique NTD states (**A-N**) were classified and reconstructed for the R95G mutant (ADP-bound) by cryo-EM. The z-slices of the three-dimensional maps display the densities of the NTDs in the ‘up’ conformation (light gray) in each state. Panel (**C**) was modified from [123,124] and panel (**D**) was adapted from [74] with permission.

The functional state of p97 is a homo-hexamer. Each subunit comprises an N-terminal domain (NTD), two tandem nucleotide-binding domains, and a short C-terminal domain [109]. The ATPase domains, denoted D1 and D2, of each subunit assemble to form two coaxially stacked rings, resulting in 12 ATPase domains per p97 complex. The NTD is linked to the D1 domain by a short loop and functions to recruit adaptor proteins and substrates. The NTD can adopt an ‘up’ or ‘down’ conformation characterized as being situated above or coplanar to the D1 ring, respectively (Figure 2A) [110,111]. Upon nucleotide binding and hydrolysis, allosteric signals originating in the ATPase domains result in large-scale domain motions of the NTD. While ATP binding to the D1 ring stabilizes the ‘up’ conformation, ADP favors the ‘down’ state. Recent studies have characterized the dynamic transitions that define the intermediate ADP-Pi-bound state, initially captured by NMR [125]. A combination of NMR, cryo-EM, and molecular dynamic simulations revealed that these transitions are governed by rotameric shifts in key residues [120]. Specifically, during ATP hydrolysis, conformational changes in active-site residues R359 and F360 reorganize intra-subunit interactions, thereby increasing flexibility in the Arg finger and sensor loops (Figure 2B). Notably, these sensor loops are situated near subunit interfaces, thereby establishing an interprotomer network where changes to intra-subunit interactions propagate to become inter-subunit signals. The sensor loops undergo a structural transition from a loop to a 3_10_-helix between the ATPγS- and ADP-bound states, with the rate of interconversion reflecting nucleotide turnover. This increased plasticity from ATP hydrolysis then propagates toward the NTD, resulting in a rotameric shift in H384 that alters the positioning of the helix with respect to the nucleotide (Figure 2B). This subsequently rotates N387, resulting in the formation of an electrostatic network that stabilizes the ‘down’ NTD conformation. The connection between adjacent active sites through the sensor loops therefore not only provides a means of co-ordinating ATPase function but also relates ATP hydrolysis with NTD motion. Sensor loop-mediated coupling between neighboring ATPase sites thus co-ordinates ATP hydrolysis with NTD motion [120]. These findings illustrate how local structural rearrangements, governed by side-chain rotamers and flexible loops, convert chemical energy into mechanical output. Moreover, they highlight the value of integrating cryo-EM and NMR to capture transient or subtle conformational changes within the active sites and at the subunit interfaces, which are essential to p97 function, particularly those that regulate adaptor binding through NTD repositioning.

The application of methyl-TROSY NMR enabled the investigation of NTD dynamics in p97 particles, revealing how disease-linked mutations affect p97 function [123,126]. These studies, which compared dynamics across p97 particles comprised solely of WT or solely of variant subunits, respectively, revealed that mutations disrupt the native ‘up’/‘down’ equilibrium of the NTD, with the extent of dysregulation correlating with disease severity [123,126]. In the ADP-bound state, the NTD chemical shifts revealed that while 0% of the NTDs in the WT complex adopted the ‘up’ position, ~50% of NTDs were in the ‘up’ position in the severe R95G variant (Figure 2C) [123,124,126]. Moreover, the NTD of mutant complexes bound to ADP displayed rapid interconversion between the ‘up’/‘down’ states (>15,000 s^−1^), in contrast with the more stable conformational behavior of the WT enzyme, likely due to disrupted interactions at the NTD–D1 interface [123,126]. Cryo-EM structures of MSP-1 mutants R155H, A232E, and R191Q further provided a structural basis for these modified dynamics [127]. These mutations resulted in 14%, 18%, and 39% of NTDs in the ‘up’ position as detected by NMR [123], respectively, and each introduced unique perturbations that disrupt native interactions in the ADP-bound state [127]. The less severe mutations, R155H and A232E, disrupted H bonding pairs between the NTD and D1 domains, imparting increased flexibility of the NTD. Conversely, the more severe R191Q mutation promoted helix formation in the NTD–D1 linker, a structural transition characteristic of the ATPγS-bound state. As a result, the mutations destabilize the ADP-bound ‘down’ state, inducing the fast exchange between the ‘up’ and ‘down’ NTD conformations [127]. In aggregate, these techniques provide a clear structural basis for the impact of mutations on homogenous p97 assemblies—the disruption of key contacts between the NTD and D1 domain via point mutations alters NTD dynamics, which in turn affects p97 function.

In addition to homogeneous p97 complexes, heterogeneous complexes can arise in heterozygous patients containing alleles of WT and disease mutations [128]. These particles consist of different proportions of WT and disease-linked mutant subunits depending on their relative expression levels. This raised questions about the role of co-operativity in p97 function and how disease mutations may reshape allosteric interactions between subunits. These inquiries have been investigated by an NMR study on heterogeneous p97 complexes containing a single, NMR-visible mutant subunit against an NMR-invisible background, prepared through ‘subunit mixing’ experiments described above [104,105]. The results demonstrated that the NTD dysregulation is composition-dependent [124]. For example, when an R95G subunit was surrounded by WT subunits, only 27% of its NTDs adopted the ‘up’ conformation in the ADP-bound state (Figure 2C). Conversely, when the R95G subunit was surrounded by a more dysregulated variant, such as the double variant R95G/T262A, the population of R95G NTDs in the ‘up’ conformation increased significantly to 82%. While the bulk NMR measurements revealed co-operativity between subunits and the amount of ‘up’ or ‘down’ NTDs [124], they were unable to provide information on the conformations of each of the subunits within individual particles. High-resolution cryo-EM structures were therefore used to investigate how co-operativity between neighboring NTDs could manifest as different structural states of a p97 complex [74]. A total of 14 unique conformational states exist for a p97 hexamer due to the two possible positions of each NTD (Figure 2D). Analysis of R95G variant complexes bound to ADP successfully identified all 14 states, enabling the derivation of a probabilistic model that related a subunit’s conformation to the states of its neighbors. Interestingly, the NTD equilibrium of the R95G variant was independent of neighboring subunits. When considered together, the NMR and cryo-EM data support that the degree of co-operativity is dependent on the composition of the p97 complexes: while co-operativity was observed between the NTD conformational states in heterogeneous p97 complexes, the homomeric, ADP-bound R95G complexes displayed little inter-subunit communication that affects the conformational equilibrium of the neighboring NTDs.

Advancements in our ability to measure transient structural fluctuations have added to a growing body of work that defines the dynamics which governs p97 function. Allosteric relationships have been noted beyond the NTD and D1 ring, with co-operativity recently being shown between the active sites of the D1 ring upon ATP hydrolysis [120]. Additionally, binding of inhibitors to the D2 ring can allosterically trap the D1 ring and NTD into a conformation that is no longer responsive to the bound nucleotide [121,122,127]. These developments have also led to a variety of new high-resolution structures investigating p97-adaptor protein complexes and substrate processing [117,122,129–134], as well as novel p97 oligomeric states [135,136]. By using methods adept at capturing these dynamics, we can begin to understand the biological role of the short-lived regulatory structures and exploit them in the search for novel therapeutics.

### ClpP

Caseinolytic protease P (ClpP) is a conserved serine protease found in bacteria, plastids, and human mitochondria. It functions as a crucial component of the proteostasis network through regulated degradation of target protein substrates [137–139]. In bacteria, ClpP has been identified as a key regulator of a variety of cellular functions, including cell division, response to heat stress, sporulation, DNA repair, and protein quality control [138,140,141]. This is accomplished via the targeted degradation of regulatory factors or damaged proteins, allowing for an adaptable response to cellular needs. Mitochondrial ClpP contributes to protein quality control within the organelle in a similar manner. Located in the mitochondrial matrix, ClpP mitigates the effects of oxidative stress through the degradation of oxidized and damaged proteins [142]. ClpP also contributes to the virulence of pathogenic bacteria as well as the survival and metastasis of cancer cells [139,142,143]. For these reasons, there is major interest in deregulating the protease as a therapeutic strategy against both antimicrobial resistance [144–146] and cancer [147,148]. Exploiting the inherent conformational plasticity and allosteric regulation of this protease system offers promise as a means of disrupting ClpP function.

**Figure 3:**
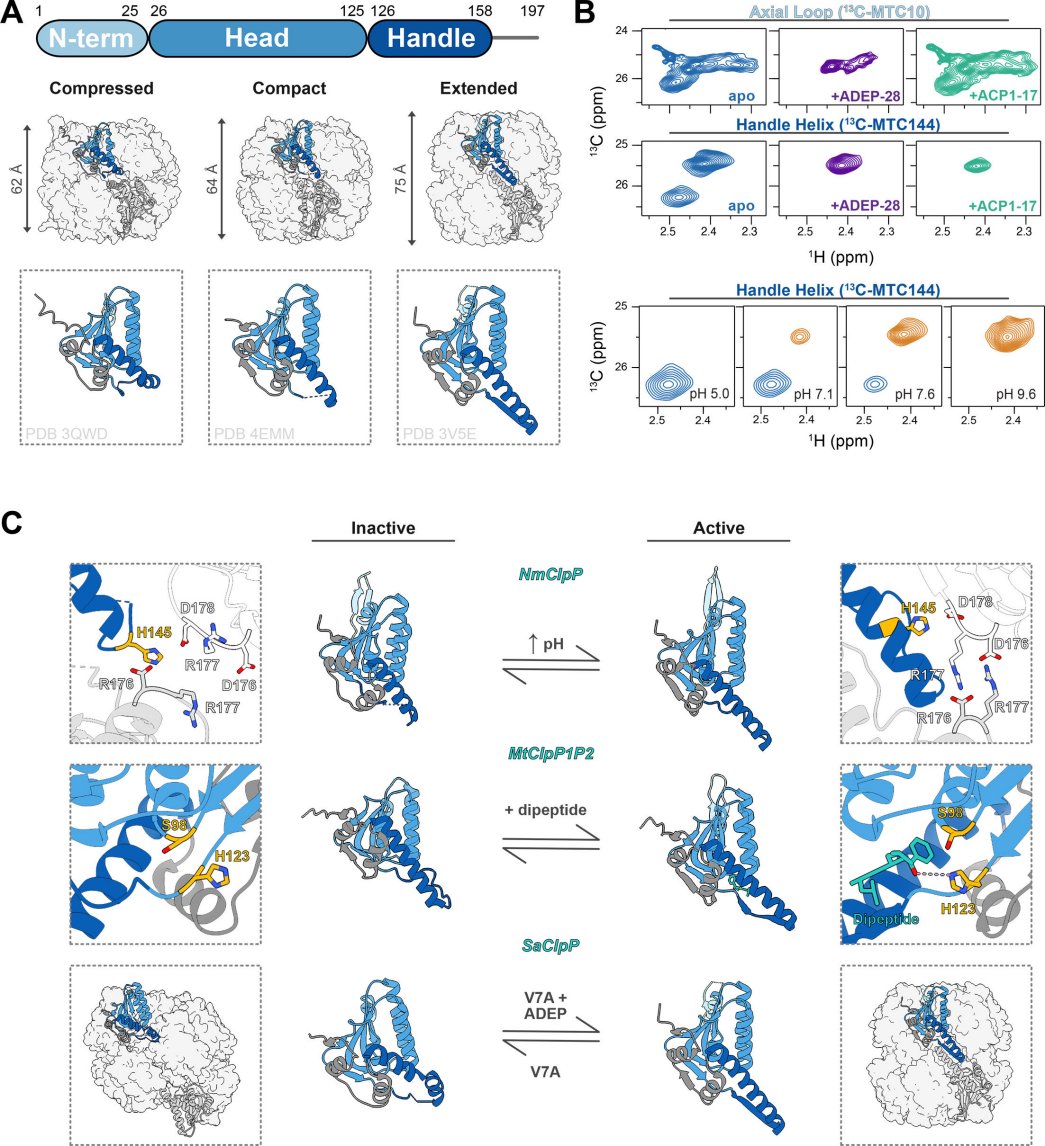
ClpP dynamics are modulated by various stimuli, with the handle helix ultimately acting as an on/off switch for activity. (**A**) (Top) Domain arrangement of a ClpP monomer, with residue numbering of ClpP expressed by *Staphylococcus aureus* ClpP (SaClpP); (middle) the ClpP tetradecamer can adopt one of three barrel heights, denoted compressed (PDB ID 3QWD [150]), compact (PDB ID 4EMM [151]), and extended (PDB ID 3V5E [152]), which is controlled by the conformation of the handle helix (bottom); (**B**) 1H-13C HMQC NMR spectra of MMTS-labeled *Neisseria meningitidis* ClpP, with single ^13^C probes at the axial loop (residue 10) and handle helix (residue 144), to monitor protein dynamics at these sites. (Top) Binding of ADEP reduces structural heterogeneity within the axial loops and handle helix, while ACP binding only affects the handle helix conformation. (Bottom) Changes in pH can also modulate the conformational states of the handle helix by altering the balance of protonation states of key residues; and (**C**) ClpP enzymes interconvert between the active extended form and inactive compact or compressed forms, as shown through cryo-EM. (Top) NmClpP activation relies on establishing salt bridges between inter-ring oligomerization sensors (D178, R177, and D176), which stabilize the handle helix and orient the catalytic residues (PDB ID 7KR2 for the inactive [153] and 6VFS for the active [154] states); (middle) activation of ClpP1P2 from *Mycobacterium tuberculosis* can be similarly accomplished through the binding of a dipeptide that stabilizes the handle helix domain (PDB ID 6VGK for the inactive [155] and 5DZK for the active [156] states); (bottom) introduction of a single point mutation to the N-terminal domain of SaClpP induces an inactive, split-ring conformation (PDB ID 6DKF for the inactive [157] state). The binding of ADEP can rescue this phenotype and promote the active ClpP conformation (PDB ID 3V5E for the active state). Panel (**B**) was adapted from [158] and [153] with permission. NmClpP, *Neisseria meningitidis* ClpP; ADEP, acyldepsipeptide.

ClpP structure is retained across all domains of life [149]. It is formed through the stacking of two homo-heptameric rings, resulting in a barrel-shaped complex with 14 catalytic sites sequestered inside the enclosure (Figure 3A). At the two axial ends of the barrel, the N-terminal residues of ClpP form gated narrow pores that restrict entry of large and folded substrates into the degradation chamber. For this reason, ClpP alone can only act upon small and unstructured substrate proteins. The binding of hexameric AAA+RPs, such as ClpX, ClpA, and ClpC, confers substrate specificity and enables the unfolding and translocation of large and folded protein substrates for degradation. In many bacteria, proteins bearing an SsrA sequence (ANDENYALAA in *E. coli*), which is added co-translationally to the C terminus of incompletely synthesized polypeptides are targeted by the ClpXP protease complex [159,160]. More recently, other recognition motifs, such as phosphorylated arginine residues, were discovered as degrons recognized by the ClpC RP in *B. subtilis* [161]. Substrates bearing phosphorylated serine or threonine residues are preferentially degraded by ClpXP in the human mitochondria [162]. Degradation of substrates may also be facilitated via adaptor-mediated tethering to the RP [163]. While RP interactions mediate access into the catalytic chamber, enzymatic activity of the complex is also regulated via conformational dynamics within the ClpP barrel.

The study of ClpP enzymes has been greatly aided by the number of high-resolution structures provided by X-ray crystallography [150,164–166]. In these structures, the head domains of each subunit assemble into the aforementioned heptameric rings, while the handle domains mediate interactions across rings to form the mature tetradecameric ClpP complexes. Notably, comparison of structures across different organisms found three states of the ClpP barrel denoted compact, compressed, or extended. These states differed in barrel height and the conformation of the handle helices (Figure 3A) [20,167]. In the compact or compressed forms, which are inactive, the handle helix is partially disordered or kinked, respectively, resulting in a misalignment of the catalytic triad. By contrast, in the active form, it is fully extended, and the catalytic residues are therefore optimally oriented for catalysis. While these static structures suggested a functional role for the conformational plasticity within the handle domain, combined NMR and cryo-EM studies have provided a more comprehensive understanding of handle helix dynamics as they relate to ClpP activity. This combined approach has definitively demonstrated that the handle domain samples various conformations in solution [158] and that these populations are influenced by factors such as pH [153,168], the binding of small molecules [155,169], and structural fluctuations within the NTD [157,158].

Methyl-TROSY NMR experiments showed that the handle helix populates different conformations in solution (Figure 3B). At neutral pH, there are two distinct peaks corresponding to the handle helix of *Neisseria meningitidis* ClpP (NmClpP), indicative of two coexisting conformers [153,170]. These conformers, later shown via cryo-EM to be the compressed (inactive) and extended (active) forms, interconverted on a slow timescale with the compressed state being preferentially populated at pH 7 [153]. This is due to the loss of intra-ring electrostatic interactions at the end of the handle helix, resulting in localized unwinding with concomitant disorganization of the catalytic triad. Transition to the active form could then be triggered by an increase in pH, thereby altering the protonation state of His145 and re-establishing a key hydrogen bond with Asp178 of the neighboring subunit. This, in turn, promoted inter-ring salt bridges, known as the oligomerization sensors, stabilizing the extended handle helix conformation (Figure 3C). Recent structural studies suggest that pH sensitivity plays a regulatory role in ClpP function by modulating the equilibrium between active and inactive conformations in response to substrate hydrolysis [168]. In a similar manner, molecules targeting the catalytic sites, including substrate mimics and competitive inhibitors, can promote the transition to the active state by stabilizing the extended conformation. Interestingly, this structural switch does not require the binding of ligands to all 14 active sites but rather to a subset of ClpP protomers, which is sufficient to promote the transition of the entire complex [155,169]. Like in NmClpP, *Mycobacterium tuberculosis* (MtClpP1P2) exists primarily in the inactive state [155]. Cryo-EM revealed that apo MtClpP1P2 adopted the compact conformation characterized by a shortened barrel height and lack of β-sheets in the handle region. However, dipeptide binding (e.g. Bz-LL) in the active site or reaction of the catalytic Ser with a covalent inhibitor promotes the inactive-to-active transition, whereby the formation of the handle β-sheets connects inter-ring subunits and promotes both the positioning of the catalytic triad and engagement of the oligomeric sensor residues. These inter-subunit allosteric effects then promote the activation of the entire complex, prompting the formation of the active conformation of the enzyme within the unoccupied subunits. Similar sub-stoichiometric activation has also been noted in the *Thermus thermophilus*, human (TtClpP and hClpP, respectively), and MtClpP1P2 ClpP systems upon engagement with a competitive inhibitor [169,171–173]. It was also found that the activation of TtClpP or MtClpP1P2 in this manner promoted the association with the respective RP of each system, TtClpX, MtClpC1, or MtClpX [169,173]. These results suggest that co-operativity extends beyond the center of the complex, linking peptidase function to distal RP-binding sites.

The dynamics of the handle helix can also be modulated by targeting the axial ends of the chamber, ~50 Å away. The association of small-molecule activators to the RP-binding sites was previously shown to be bactericidal through the allosteric activation of the ClpP complex, thus promoting unregulated substrate degradation within the cell [174]. In NMR spectra, the association of these compounds, namely acyldepsipeptides (ADEPs) or activators of self-compartmentalizing proteases 1, stabilized the extended conformation of the handle helix and promoted the active state of NmClpP [158]. Moreover, the binding of ADEPs to the MtClpP1P2 complex increased affinity for the activating dipeptides (e.g. Bz-LL), lowering the EC_50_ of the activating dipeptides [155]. ADEP binding affected not only the dynamics of the handle helix but also those of the axial N-terminal loops. These loops, which are responsible for restricting access into the degradation chamber, are highly flexible, conformationally heterogeneous structures as established by NMR measurements [157,158]. Heterogeneity reflected within the NMR spectra decreased upon ADEP binding in both the MtClpP1P2 [155] and NmClpP [158] systems, indicating a rigidification of the region—a fact that was corroborated by high-resolution cryo-EM and X-ray crystallography structures.

Outside of small-molecule effectors, point mutations within the NTD can also influence dynamics within the handle helix [157]. Investigations into the NTD using NMR noted that mutation of a hydrophobic cluster (Pro5, Val7, and Ile20), namely a V7A substitution, resulted in complex-wide CSPs and eliminated catalytic activity of the *Staphylococcus aureus* ClpP (SaClpP) complex [157]. The structural basis for these large-scale changes was elucidated using cryo-EM, which revealed that the V7A mutation induced the formation of a split-ring conformation characterized by two 20-Å-wide pores in the sides of the barrel (Figure 3C). This arose from asynchronous conformations within the heptameric rings leading to the differential elevation of subunits. To maintain the oligomerized state, six of the subunits adopted an extended-like conformation, where the N-terminal end of the handle helix slightly unfolds, while the seventh subunit adopted the compact conformation with a kinked handle helix. Moreover, the V7A mutation induced unfolding of the NTD. NMR and activity analyses of mixed SaClpP tetradecamers found that a single mutated subunit within the tetradecamer was sufficient to unfold all NTDs within the complex and abolish enzymatic activity. This indicates a high degree of co-operativity across the NTDs and further establishes a relationship between the dynamics of this domain and catalytic function. Interestingly, while the binding of ADEPs to V7A SaClpP partially restored peptidase function and promoted an extended-like state within subunits, it was not sufficient to restore NTD structure [157].

From these orthogonal views of ClpP, it is clear that inter- and intra-subunit co-operativity regulate ClpP function with the handle helix acting as an on/off switch for activity. Building off foundational X-ray crystallography work, cryo-EM is now providing high-resolution structural detail of the more dynamic components of the ClpP system, which previously resisted crystallization [175,176]. This has led to insights on RP-bound complexes [154,177,178], substrate recognition and processing [179,180], and interactions between ClpP rings [181]. A more exhaustive analysis on the findings of cryo-EM studies related to ClpP regulation can be found in a recent review [170]. As the number of high-resolution structures has increased, so too has the number of studies analyzing ClpP dynamics [172]. The result is a more comprehensive understanding of how plasticity and allostery regulate ClpP function, and how we can exploit these properties in the treatment of human diseases.

### DegP

Degradation of periplasmic proteins (DegP) or protease Do, is a periplasmic serine protease found in *E. coli* and other Gram-negative pathogens that helps with maintaining bacterial proteostasis [182,183]. It belongs to the HtrA family of proteins, which function both as ATP-independent proteases and chaperones [184]. Outside of contributing to cellular proteostasis, the chaperone function of DegP has also been linked to bacterial pathogenesis as it traffics outer membrane proteins [185] and virulence factors through the periplasmic space [186–188]. Indeed, *degP* knockouts attenuate bacterial virulence at 37°C and are lethal at higher temperatures [189]. DegP is, therefore, a promising target for drug therapies against bacterial infections, which necessitates a comprehensive understanding of the dynamics that govern its function.

**Figure 4:**
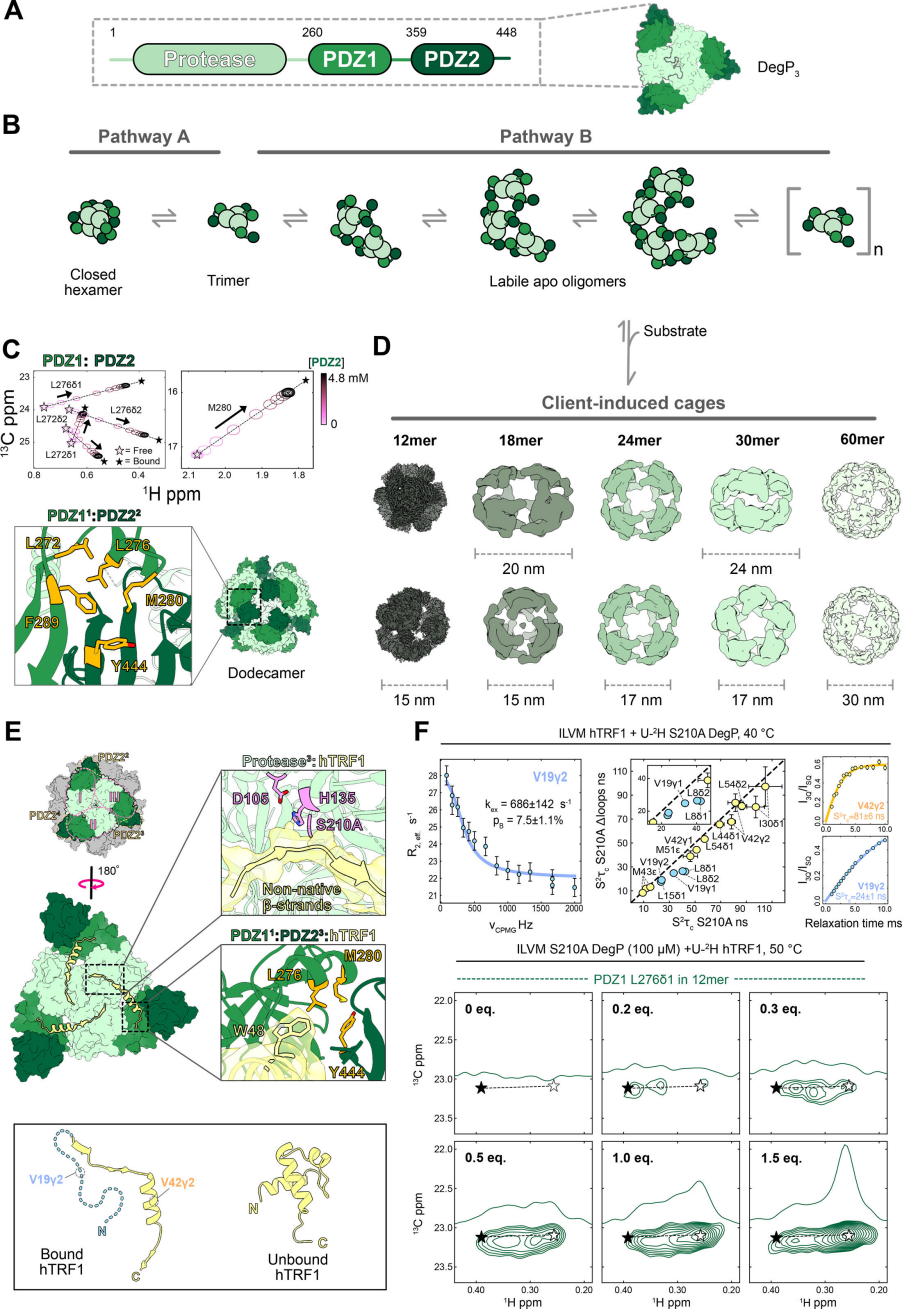
Exploring the oligomeric landscape of the cage-forming DegP protease-chaperone using methyl-TROSY NMR and cryo-EM. (**A**) (Top) Domain arrangement of a DegP monomer (PDB ID 8F0A [192]); (**B**) competing cellular stress-dependent pathways govern the self-assembly landscape of apo DegP. One of these leads to the closed hexamer, while the other gives rise to an ensemble of rapidly exchanging, loosely associated cage-like species; (**C**) methyl-TROSY NMR-based titration series monitoring PDZ1 methyl group resonances that reveals the higher order unbound oligomers form through the same inter-trimer PDZ1*^i^*–PDZ2*^j^* interactions (*i* and *j* denote domains from separate trimers, *i*≠*j*) that stabilize the substrate-bound DegP cages (PDB ID 8F0U [192]); (**D**) cryo-EM structures of an array of DegP cages that are adopted to accommodate substrates of different sizes (EMDB IDs EMD-28781 (12mer), EMD-28800 (18mer), EMD-28801 (24mer), EMD-28806 (30mer), EMD-28808 (60mer) [192]); and (**E**) analysis of client–DegP interactions using cryo-EM (top) revealed that hTRF1 is remodeled upon binding to DegP, yet it also retains native-like secondary structure (bottom) (PDB ID 1BA5 (unbound substrate), 8F0U (substrate-bound 12mer) [192]). This positions hTRF1 for cleavage via the DegP protease domains (top right) and leads to interactions with the PDZ1*^i^* and PDZ2*^j^* domains (prime denotes a domain from a different trimer) that stabilize the 12mer cage; (**F**) measurements of μs-ms and ps-ns hTRF1 dynamics within the 12mer cage via CPMG (top left) and relaxation-violated NMR experiments (top middle and right) reveal that its N-terminal half rapidly exchanges between at least two conformations (top left) and its methyl groups are less restricted than the C-terminal bound portion (top middle and right). Furthermore, its N-terminal methyl groups are slightly more mobile in the absence of DegP’s long protease domain loops (see correlation plot), indicating that weak attractive or repulsive interactions occur in the cage interior. A titration of apo DegP with hTRF1 (bottom), monitoring the PDZ1 domain L276δ1 methyl group signature through methyl-TROSY NMR established co-operativity in 12mer cage formation and enabled tracking of substrate occupancy. The black and white stars, respectively, correspond to the chemical shifts of L276δ1 in apo PDZ1 domains of partly bound trimers within cages (black) and in bound PDZ1 domains of saturated trimers within cages (white). The resonances appearing between these positions correspond to partly ligated cages. The black star frequency was obtained from the titration of the PDZ1 domain with the PDZ2 domain in panel C, where the L276δ1 methyl resonance shifts along the pink (apo) to white (bound) star trajectory. Substrate addition generates stable ligated cages as indicated by the resonances appearing at and between the black and white stars. Panel (**C**) was adapted from [193], and panel (**E**) was adapted from [66] with permission. hTRF1, Human telomere repeat binding factor 1.

HtrA protein subunits contain one N-terminal serine protease domain and at least one C-terminal PDZ domain. They form trimers as their fundamental oligomeric unit, which are stabilized through interactions between the protease domains of the constituent monomers. The PDZ domains typically recognize disordered C-terminal sequences of substrate proteins [190,191]. In contrast with most HtrA members, DegP monomers contain two C-terminal PDZ domains, referred to as PDZ1 and PDZ2 (Figure 4A) [194]. Here, PDZ1 binds to DegP substrates, while PDZ2 mediates interactions with other trimers rather than participating in substrate interactions (described in depth below). The flexible linker regions between the domains within DegP monomers impart remarkable plasticity that facilitates the adoption of various higher order oligomeric assemblies [194–196].

Crystallography studies provided some of the first high-resolution details of these DegP assemblies [42,194,195]. In the apo state, DegP exists as a hexamer formed through the stacking of two trimers in a ‘face to face’ manner where the active sites are within the core of the complex [194]. It was found that the hexamer adopts either ‘closed’ or ‘open’ conformations, defined by the accessibility of the complex lumen. In the closed form, the assembly is stabilized via protease*^i^*-protease*^j^* (*i* and *j* denote domains from separate trimers, *i* ≠ *j*) and PDZ1*^i^*–PDZ1*^j^* interactions. In this case, the proteolytic sites are fully sequestered in the hexamer interior, leading to the assignment of this form as the cellular resting state of the protein. Conversely, the open hexamer is formed through partial separation of the trimers and increased exposure of the PDZ1 domains that together grants access to the central cavity. The interconversion between these hexameric forms was thought to be important for regulating spurious proteolysis and initial substrate docking to the PDZ1 domains [194]. Later structural studies, including crystallography and cryo-EM-based approaches, revealed that substrate engagement triggers a reorganization of the inter-trimer contacts and the adoption of new, trimer-based oligomeric states [42,195,197]. These investigations found higher order cage assemblies consisting of either 12 or 24 monomers (4 or 8 trimers, respectively) that are stabilized through interactions between PDZ1*^i^* and PDZ2*^j^* domains. Moreover, these cage species were shown to encapsulate bound substrates for proteolysis. These striking foundational structural studies of both apo and client-engaged complexes led to further questions about the trimer assembly mechanism underlying cage formation, as well as the full scope of oligomeric species accessible to DegP.

Subsequent investigations of DegP using solution NMR in combination with hydrodynamic methodologies revealed that DegP adopts an array of interconverting oligomers, mediated by trimers, in the absence of substrate [193]. Interestingly, trimer assembly was shown by concentration- and temperature-dependent dynamic light-scattering measurements to occur via two competing pathways, A or B. Pathway A is dominant at low temperatures and leads to a species that was assigned as the canonical closed hexamer (Figure 4B). Conversely, pathway B is populated at high temperatures and is highly sensitive to DegP concentration and ionic strength. It consists of an ensemble of cage-like species that, similar to the substrate-engaged structures, are supported by PDZ1*^i^*–PDZ2*^j^* domain contacts (Figure 4C). However, without substrate, the PDZ1*^i^* and PDZ2*^j^* domains only weakly associate, leading to a broad distribution of oligomers rather than tightly assembled, discrete cage structures. Methyl-TROSY NMR-based experiments afforded an estimate of the exchange rate between the pathway B oligomers of ~1800 s^-1^. It was postulated that this rapid apo-oligomer interconversion may be important for DegP to quickly respond to insults to the bacterial periplasm. Inter-trimer contacts between L272*^i^*, L276*^i^*, and M280*^i^* of the PDZ1*^i^* domains and Y444*^j^* of the PDZ2*^j^* domains were found to be critical to the apo-oligomer formation, similar to substrate-bound cages. This implied that the oligomeric states adopted in the substrate-bound form are sampled to some extent by apo DegP; however, the interactions formed are not as extensive as those in the substrate-bound structures [193,198,199]. It was also shown that M280 is a temperature-sensitive probe involved in heat-induced dissociation of the hexamer populated at low temperatures [182], as it undergoes a temperature-dependent conversion between two conformations. Interestingly, it also appeared that PDZ1*^i^*–PDZ2*^j^* interactions stabilize the low-temperature hexamer through M280*^i^* and Y444*^j^*, rather than PDZ1*^i^*–PDZ1*^j^* contacts [198]. This indicated that the pathway A hexamer is structurally somewhat different from the closed hexamer found via crystallography, which is stabilized in part by PDZ1*^i^*–PDZ1*^j^* interactions [194]. Together, these findings suggest that pathway A may function as a reservoir for DegP trimers, which are released under cellular stress. It is important to note, however, that at periplasmic DegP and salt concentrations and 37°C, pathway A is only sparsely populated [192,193], raising the question of its significance in human pathogens. Under these conditions, most of the DegP trimers were shown to reside within oligomers in pathway B. Regardless, solution-based NMR studies definitively revealed that the energy landscape of unbound DegP gives rise to a broadly populated ensemble of pre-organized states that is tuned by cellular stress conditions and is poised for substrate capture. Substrate proteins rapidly remodel this oligomer network yielding the classical cage complexes.

Other recent studies of DegP focusing on understanding its interactions with substrates using cryo-EM have also uncovered new oligomeric states [192]. It was revealed that many different cage assemblies can be formed beyond the canonical 12- and 24-mers identified in the presence of peptides or disordered proteins [195]. To further explore how DegP responds to substrates of increasing size, the authors used a series of chimeric protein constructs consisting of a C-terminal DegP affinity tag and an N-terminal ‘domain’ corresponding to a folded protein [192]. For the affinity tag, the DNA-binding domain of human telomere repeat binding factor 1 (hTRF1) was chosen as it represents a model client protein that interacts tightly with DegP. Through cryo-EM paired with dynamic light scattering measurements, a correlation between the size of the N-terminal portion of the substrates and the scale of the DegP cages that are formed was measured (Figure 4D) [192]. It was found that small substrates (<10 kDa) resulted in the previously described tetrahedral, dodecameric cages [42,195,197], while larger ones induced a distribution of cages. For example, while the binding of a ~19-kDa client resulted in largely dodecameric structures, it also induced trigonal bipyramidal 18-mers and the well-known 24-mer cages, which represented roughly 12% and 5% of the total particles, respectively. Association with a ~28-kDa client protein resulted in mostly 24-mers and pentagonal bipyramidal 30-mers. It is important to note that 18-mers and 30-mers were found to form in an earlier study of DegP binding to a fragment of lysozyme through analytical ultracentrifugation experiments [196], though structural and biophysical analyses focusing on these were not performed, as was the case in [192]. Finally, binding of a ~33-kDa client induced the largest cage assemblies, including 30-mers, icosahedral 60-mers, and, strikingly much larger amorphous species approximately 50–100 nm in diameter that could easily span the periplasmic space. It was not clear why this relatively small increase in substrate size relative to the ~28-kDa client led to the formation of 60-mer and larger cages. It is likely that other properties of substrates in addition to their size play a role in the scale of the cages formed. For example, charge repulsion may make it unfavorable for the ~33-kDa substrates to be near one another, resulting in the inflated assemblies. Notably, the structures that were modeled for each of the 12-, 18-, 24-, 30-, and 60-mers demonstrated that they all form via the inter-trimer PDZ1*^i^*–PDZ2*^j^* domain interactions, supported by the extensible inter-domain linkers of the monomers. These investigations highlighted the versatility of the trimeric unit in adopting polyhedral assemblies of varying sizes to accommodate the many different substrates that DegP encounters in the periplasm.

Along with providing evidence of incredibly large and dynamic DegP cage assemblies, cryo-EM also revealed important new details regarding DegP–client interactions [192]. By further analyses of the dataset collected for DegP in the presence of hTRF1, leading to a 2.6 Å map of a bound 12-mer cage, Harkness et al. found that the C-terminal half of the client was engaged by the PDZ1 domain of a given monomer and the protease domain of the adjacent protomer within the same trimer in the counterclockwise direction. This generated a propeller-like arrangement of the three substrate chains within a trimer (Figure 4E, top). The bipartite binding mode in which bound substrates stretch across adjacent subunits confirmed what was initially suggested by crystallography studies where only partial electron density was found for a substrate within the protease and PDZ1 domains [42,196]. In addition, the substrate was not as unfolded as previously thought and instead adopted a mixture of native and non-native structure. Interestingly, the client retained a nearly complete native α-helix spanning the protease^i^ and PDZ1^i^ domain binding sites, which was partly unwound at its C-terminal end to generate a non-native β-strand that extended a β- sheet in the PDZ1^i^ domains (Figure 4E, top and bottom). In doing so, the substrate contributed a Trp and Met residue to the inter-trimer hydrophobic clusters formed by the PDZ1^*i*^ and PDZ2^*j*^ domains of trimers in the 12-mer, further stabilizing the cage assembly in which it ultimately would be degraded. In the protease domain binding site, the native α-helical structure of the substrate was remodeled into two non-native β-strands leading to a bent, V-shaped conformation. This complemented two β-sheets of the protease domain, positioning the backbone of the substrate near the catalytic Ser for proteolysis (Figure 4E, top). These cryo-EM analyses were, however, unable to provide information on the structure of the N-terminal segment of the client, likely owing to its unfolding and concomitant flexibility within the center of the 12-mer.

Further studies of this complex leveraging methyl-TROSY NMR demonstrated that the C-terminal half of hTRF1 exists in multiple, slowly exchanging bound conformations that were not captured by cryo-EM [192]. Through methyl CPMG experiments [200], it was also shown that the N-terminal half existed as a dynamic disordered ensemble within the DegP cage, exchanging between at least two conformations with a rate constant k_ex_ of ~700 s^-1^ (Figure 4F, top left). This was further supported by experiments reporting on methyl group amplitudes of motion [80] throughout the hTRF1 chain, where N-terminal methyl groups had smaller order parameters relative to the C-terminal bound portion (Figure 4F, top middle and top right), which indicated that they are more mobile. These experiments also revealed that the N-terminal methyls experienced transient interactions with the long DegP protease domain loops in the interior of the 12-mer, which slightly restricted their motions (Figure 4F, top middle) [66]. The weak loop interactions with the substrate used in this study did not appear to influence proteolytic activity. However, with other substrates where the strength of these interactions may be increased, they could potentially modulate proteolysis, e.g. through biasing product release or stabilizing the bound state of the protease domains. Another intriguing finding from this NMR study emerged from a titration of DegP with substrate, where binding of only one or two of these substrates to a given trimer was sufficient to drive cage formation (Figure 4F, bottom). This co-operative cage assembly was in agreement with earlier work implementing a disordered protein segment [196], emphasizing that trimers which are partially saturated undergo an allosteric conformational change to form cages that are only somewhat occupied [66,196]. Saturation of the cage trimers with substrate was subsequently shown to be necessary for proteolysis [66]. Taken together, these studies provide a possible explanation for the switch between DegP’s protease vs. chaperone functions, where partly engaged trimers in cages hold substrates, potentially allowing them to refold, while those that become fully bound cleave them.

The combination of high-resolution cryo-EM and solution NMR studies in application to DegP is perhaps one of the most remarkable examples of the synergy between these two methods. Unlike the other protein systems presented here, DegP adopts a continuum of oligomeric structures in apo and substrate-bound forms, the base trimer units of which are highly allosteric. Multiple levels of co-operativity govern DegP function in substrate binding, oligomerization, and proteolytic activation. The usage of cryo-EM and NMR has proven essential to dissecting each of the layers of DegP dynamics and the structures of the states involved. Solution NMR has also been invaluable to understanding the client interactions and PDZ1 domain function of HtrA2 [201,202]—a mitochondrial homolog which has garnered interest for its implication in arthritis [203] and Parkinson’s [204]. Further studies of bacterial HtrA proteins, for instance, *E. coli* DegS [205], *M. tuberculosis* PepD [206], and *C. jejuni* HtrA_CJ_ [207], are expected to similarly benefit from applications of these methods.

## Conclusions

Proteostasis is essential in all kingdoms of life, and the biomolecular machines maintaining it often form large oligomeric complexes which exhibit co-operative behavior. Indeed, it has been estimated that approximately 70% of human proteins adopt oligomeric assemblies, in which allostery is pervasive [89]. A deeper understanding of inter- and intra-subunit allosteric communication in these biomolecular machines therefore might offer promising strategies for functional modulation in diseases where these complexes are mutated. Many of the outstanding questions regarding the mechanisms of action of the protein degradation machinery often stem from the fact that these systems are highly dynamic and heterogeneous, which makes them challenging to characterize structurally. It is likely that the mechanisms of substrate tagging, recognition, and proteolysis involved are not amenable to elucidation using any single method. This review highlights the need for integration of different structural biology tools that are capable of probing structural heterogeneity and plasticity. The examples described herein demonstrate how a combined approach involving methyl-TROSY NMR and cryo-EM, built upon the methodological developments in both techniques over the past roughly two decades, can effectively overcome some of these challenges and provide highly complementary and unique insights. Although we have emphasized the strength of pairing NMR and cryo-EM, other techniques have emerged for analyzing structural heterogeneity that are similarly formidable when joined with orthogonal structural biology methods. For example, mass spectrometry (MS)-based approaches such as H/D exchange-MS [208] and native MS [209] have also become well suited to probing dynamics and scarce conformations, especially when combined with cryo-EM [210,211] and X-ray crystallography [172]. Furthermore, advances in single-molecule measurements have provided unique mechanistic insights inaccessible to methods that report on solution ensemble averages [212–215], which complement high-resolution structural data. Computer simulations leveraging increasingly powerful hardware and ever-improving force fields are fast approaching biologically relevant timescales, and when combined with experimental approaches, these promise to drive rapid advances in our understanding of biomolecular complexes [216–218]. The synergies that result from joining these methodologies will be key to answering the countless outstanding questions in the exciting area of allostery in large protein complexes.

## Abbreviations

AAA+: ATPases associated with diverse cellular activities
ACP1s: activators of self-compartmentalizing proteases 1
ADEPs: acyldepsipeptides
CPMG: Carr-Purcell-Meiboom-Gill
CSPs: chemical shift perturbations
CTD: C-terminal domain
ClpP: caseinolytic protease P
DegP: degradation of periplasmic proteins
HtrA: high-temperature requirement A
IBMPFD: Inclusion body myopathy with Paget disease of bone and frontotemporal dementia
MMTS: methyl methanethiosulfonate
MS: mass spectrometry
MSP1: multisystem proteinopathy disorder
NMR: nuclear magnetic resonance
NTD: N-terminal domain
NmClpP: *Neisseria meningitidis* ClpP
RP: regulatory particle
SaClpP: *Staphylococcus aureus* ClpP
VCP: Valosin-containing protein
WT: wildtype
cryo-EM: electron cryomicroscopy
hTRF1: human telomere repeat binding factor 1
methyl-TROSY: methyl-transverse relaxation optimized spectroscopy
